# Trusted residents and housing assistance to decrease violence exposure in New Haven (TRUE HAVEN): a strengths-based and community-driven stepped-wedge intervention to reduce gun violence

**DOI:** 10.1186/s12889-023-15997-x

**Published:** 2023-08-14

**Authors:** Guangyu Tong, Virginia T. Spell, Nadine Horton, Thomas Thornhill, Danya Keene, Christine Montgomery, Donna Spiegelman, Emily A. Wang, Brita Roy

**Affiliations:** 1 Department of Biostatistics, Yale School of Public Health, New Haven, CT USA; 2 Center for Methods of Implementation and Prevention Science, Yale School of Public Health, New Haven, CT USA; 3 Urban League of Southern Connecticut, Stamford, CT USA; 4 Yale School of Medicine, New Haven, CT USA; 5 SEICHE Center for Health and Justice, Yale School of Medicine, New Haven, CT USA; 6 Public Health Modeling, Yale School of Public Health, New Haven, CT USA; 7 Department of Social and Behavioral Health, Yale School of Public Health, New Haven, CT USA; 8 Clifford Beers Guidance Clinic, New Haven, CT USA; 9 Department of Social Work, Southern Connecticut State University, New Haven, CT USA; 10 Department of Population Health, New York University Grossman School of Medicine, New York, NY USA; 11 Department of Medicine, New York University Grossman School of Medicine, New York, NY USA

**Keywords:** Gun violence, Structural racism, Collective wellbeing, Housing stability, Incarcerated population, Stepped wedge design, Multi-component implementation, Community-based intervention, Learn as you go

## Abstract

**Background:**

We describe the rationale and study design for “**TRU**sted **r**Esidents and **H**ousing **A**ssistance to decrease **V**iolence **E**xposure in **N**ew Haven (TRUE HAVEN),” a prospective type 1 hybrid effectiveness/implementation study of a multi-level intervention using a stepped wedge design. TRUE HAVEN aims to lower rates of community gun violence by fostering the stability, wealth, and well-being of individuals and families directly impacted by incarceration through the provision of stable housing and by breaking the cycle of trauma.

**Design:**

TRUE HAVEN is an ongoing, multi-level intervention with three primary components: financial education paired with housing support (individual level), trauma-informed counseling (neighborhood level), and policy changes to address structural racism (city/state level). Six neighborhoods with among the highest rates of gun violence in New Haven, Connecticut, will receive the individual and neighborhood level intervention components sequentially beginning at staggered 6-month steps. Residents of these neighborhoods will be eligible to participate in the housing stability and financial education component if they were recently incarcerated or are family members of currently incarcerated people; participants will receive intense financial education and follow-up for six months and be eligible for special down payment and rental assistance programs. In addition, trusted community members and organization leaders within each target neighborhood will participate in trauma-informed care training sessions to then be able to recognize when their peers are suffering from trauma symptoms, to support these affected peers, and to destigmatize accessing professional mental health services and connect them to these services when needed. Finally, a multi-stakeholder coalition will be convened to address policies that act as barriers to housing stability or accessing mental healthcare. Interventions will be delivered through existing partnerships with community-based organizations and networks. The primary outcome is neighborhood rate of incident gun violence. To inform future implementation and optimize the intervention package as the study progresses, we will use the Learn As You Go approach to optimize and assess the effectiveness of the intervention package on the primary study outcome.

**Discussion:**

Results from this protocol will yield novel evidence for whether and how addressing structural racism citywide leads to a reduction in gun violence.

**Trial registration:**

ClinicalTrials.gov Identifier: NCT05723614. Registration date: February 01, 2023. Please refer to https://clinicaltrials.gov/ct2/show/NCT05723614 for public and scientific inquiries.

**Supplementary Information:**

The online version contains supplementary material available at 10.1186/s12889-023-15997-x.

## Background

### Introduction

Community gun violence kills more than 15,000 people in the U.S. each year, [1] and it is the underlying cause of the highest number of years of potential life lost among Black men under 55 years of age [2]. Gun assaults concentrate within neighborhoods, usually those where more racial and ethnic minorities live [3]. At the root of these neighborhood conditions are the effects of generations of structural racism, including redlining policies and mass incarceration, which contribute to systemic disinvestment in and disruption of community bonds through the lack of home ownership and diminished neighborhood cohesion [4–7]. Living in violence-endemic neighborhoods – whether or not one is personally victimized – is associated with chronic stress, poor cognitive performance, and poor health outcomes, due at least in part to the persistent experience of trauma [3]. Health sequelae of gun violence are, in turn, amplified along racial lines [8–10]. For decades, gun violence has been associated with high rates of unemployment, low educational achievement, poor birth outcomes, and high rates of chronic health conditions [6, 7, 11].

Because high rates of gun violence are associated with negative systemic, community-level policies (i.e., redlining and restrictions post-incarceration) and their resultant social context (e.g., structural racism), we hypothesize that a counteracting positive, systemic community-level approach is required to change the context within which intergenerational cycles of discrimination, trauma, incarceration, and gun violence persist. As such, we apply two complementary, positively-framed theoretical frameworks, (1) collective well-being and (2) assets-based community development, to upend the negative effects of structural racism that has resulted in violence-endemic neighborhoods [12–16]. Utilizing a positively-framed, strengths-based approach to the design of community-based interventions is more likely to result in feasible, effective, and sustainable programs [17]. The collective well-being framework describes four categories of fundamental community conditions (i.e., environmental, psychosocial, systems and economic) that influence five domains of community-level well-being (vitality, opportunity, connectedness, contribution and inspiration) [18].

Indeed, emerging literature has shown that community-led programs that aim to improve community conditions are associated with better community health and reduced gun violence [15]. On average, ten more such community-based programs or organizations in a city with a population of 100,000 could causally reduce the homicide by 9% and violent crime by 6% [14]. Improving housing conditions and developing vacant land can also help increase community connectedness, perceived neighborhood safety and reduced stress among community members. Findings from our recent qualitative study in the New Haven area also suggest that increasing home ownership and providing affordable mental health resources could help community members navigate life problems and potentially reduce their risk of involvement in gun-related activities [19].

Thus, with the overall goal of reducing gun violence at the community-level, we applied the novel, positively-framed, evidence-based framework of collective well-being [16] to design a multi-level intervention with three primary components: (1) housing stability (paired with financial education, individual level), (2) training trusted community members in trauma-informed counseling (neighborhood level), and (3) policy change to address structural racism (city/state level). We hypothesize that this set of interventions will systematically strengthen four collective well-being domains (Vitality, Opportunity, Connectedness, and Contribution) through bolstering two of the fundamental community conditions (Psychosocial and Economic) to create the context that enables well-being among neighborhoods most affected by community gun violence.

**Fig. 1:**
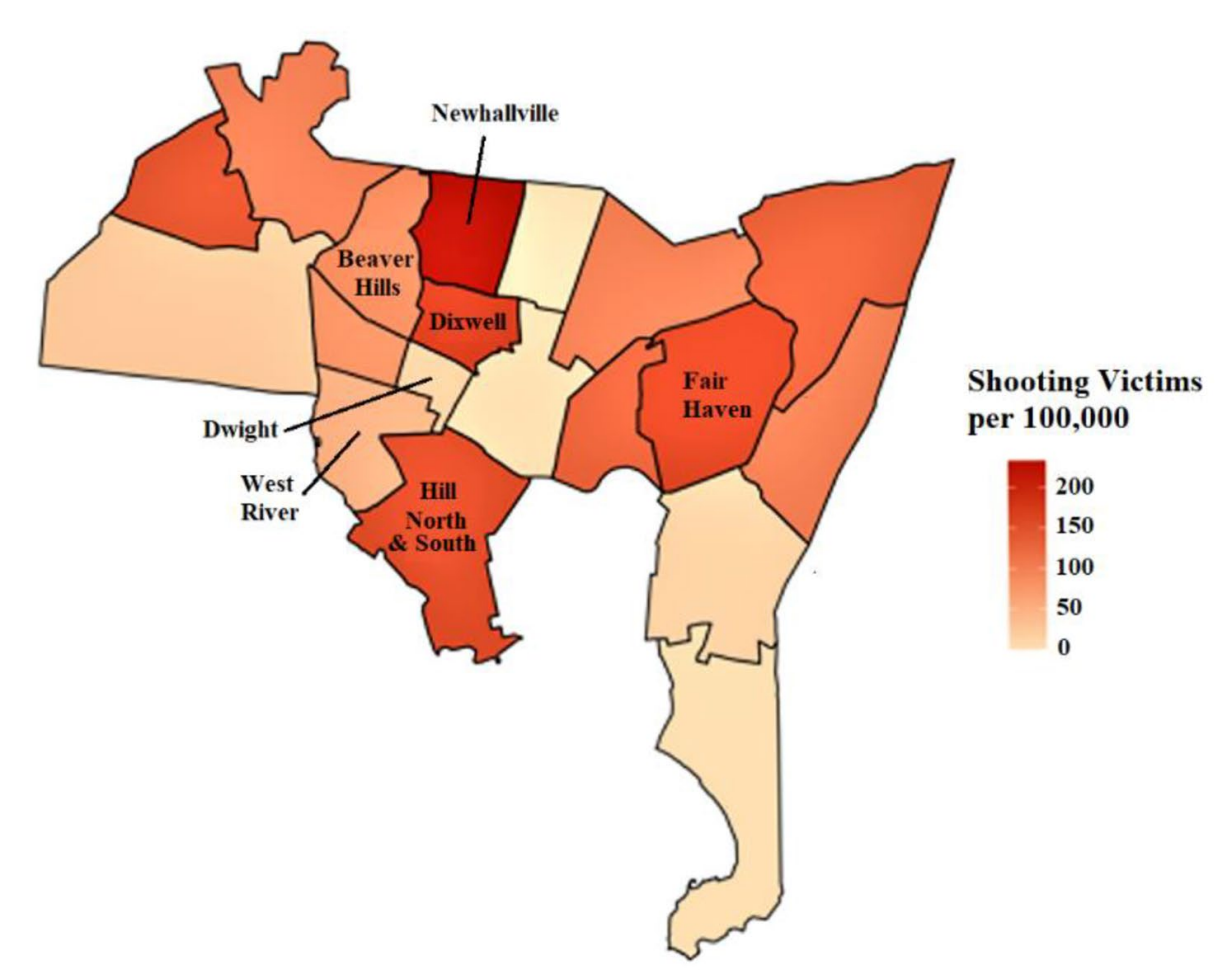
New Haven neighborhood shooting victims per 100,000 in the year of 2020

Our approach differs from most existing strategies in four major ways: (1) it deviates from individual-level approaches that focus on supporting the victim in isolation or on remediating the perpetrator of gun violence, [3, 12, 20] recognizing that the family and community context has greater long-term influence on behaviors and outcomes, [13]. (2) it applies a multi-level and multi-system approach because single-system approaches like expanding housing opportunities or access to mental healthcare alone are insufficient to sustain positive outcomes and to counteract other unfavorable community-level forces in reducing gun violence, (3) it engages institutions that have benefited from structural racism to actively re-invest in these communities, and (4) it leverages existing community assets and systematically strengthens community capacity and collective efficacy across the domains of collective well-being. We optimize and test this intervention package as they are actively delivered across each of the six neighborhoods (see Fig. 1) with among the highest rates of gun violence in New Haven using a stepped wedge trial design.

### Rationale for the study

The rationale for our study design is guided by our existing research experience and evidence acquired by our community-academic team that has been collaborating on efforts to reduce community gun violence for more than a decade. After a sharp rise in homicide rates in 2011 in New Haven, CT, our team formed with the aim of developing community-driven approaches to mitigating the incidence and effects of chronic gun violence, using an assets-based approach to leverage and enhance existing community resources and strengths (the assets) in the intervention design [13, 21]. Over the next several years, our group found that neighborhood cohesion, or the trusting network of relationships and shared values and norms of residents in a neighborhood, was associated with less exposure to violence. We thus developed strategies to enhance neighborhood cohesion in two low-income, primarily Black neighborhoods historically targeted by redlining, mass incarceration, and unequal access to healthcare, socially disadvantaging these neighborhoods [12]. Key community partners implemented complementary strategies to support and mentor youth, provide social services to those engaged in perpetration of violence, diffuse tensions, and support victims of gun violence and their families. While some progress was made, with a decline in rates of gun violence over the next five years, rates plateaued and began to rise starting in 2019. Our team has recognized that larger-scale, community-wide, systemic interventions are needed to make further progress and to sustain progress as rates of gun violence rapidly increased in 2020.

As such, we used a systems science approach to map community factors that collectively result in high rates of gun violence and to identify leverage points within this system that are likely to result in sustained reductions in community gun violence. Using a participatory approach, our multi-stakeholder, community-academic team including representatives from housing, education, law enforcement, healthcare, academics, community leaders, youth programs, mental health, crisis outreach, and those with a history of incarceration co-designed a causal loop diagram (i.e., a diagram that depicts how each community factor contributing to gun violence affects each of the others) to better understand the complex interplay of these and other community factors that allow gun violence to persist. Using the causal loop diagram, our team identified five key leverage points that together are likely to reduce the incidence of gun violence and mitigate its community-wide effects: socioeconomic status, mental well-being, social cohesion, criminal-legal system, and gang influence. In addition, data from in-depth qualitative interviews performed among residents of New Haven neighborhoods with high rates of gun violence were used to triangulate findings and prioritize key leverage points to act on. Emergent themes from these qualitative data included community building, strengthening long-term residence and homeownership, and improving access to mental healthcare as key mechanisms for reducing community firearm violence [19]. As such, our final set of interventions was solidified to include strengthening housing stability and mental well-being.

### Objectives and hypotheses

Among communities with well-being damaged by gun violence, our study aims to compare effectiveness of TRUE HAVEN vs. conditions as usual on the incidence of gun violence at the neighborhood level. We hypothesize TRUE HAVEN will be associated with lower incident gun violence compared with conditions as usual. Second, we will compare the effect of TRUE HAVEN vs. conditions as usual on secondary outcomes including social cohesion of the neighborhood, and among participants enrolled in the housing stability component, changes in their self-efficacy, perceived health and well-being, and financial security. We hypothesize that the intervention will lead to improvement in these outcomes. Lastly, for each intervention component, we hypothesize the following: (1) pairing rigorous financial education with financial support for home ownership and rental assistance will increase families’ housing stability and neighborhood cohesion, changing the neighborhood context and decreasing gun violence; (2) training trusted community members in trauma-informed counseling will provide needed psychosocial support for families and communities affected by gun violence, heighten awareness of community members affected by trauma, destigmatize accessing mental healthcare, and improve access to mental healthcare, thereby breaking the cycle of trauma and violence; and (3) convening a coalition of decision-makers and influencers at the city and state level to address structural and policy barriers that prohibit achieving housing stability and accessing mental healthcare, thereby actively dismantling structurally racist policies and facilitate components (1) and (2).

## Methods

### Overall design

The purpose of TRUE HAVEN is to assess the feasibility of implementing a city-wide intervention to address structural racism, and if so, to evaluate the extent to which it reduces the incidence and effects of community gun violence and creates the enabling conditions for historically marginalized populations to thrive. Funded by the National Institute on Minority Health and Health Disparities (NIMHD), TRUE HAVEN is a hybrid type 1 effectiveness-implementation stepped wedge trial [22–24] enrolling participants living in one of six participating neighborhoods (see Fig. 1). The staggered roll-out of the stepped wedge designs is useful for rolling out and maintaining complex multi-level community-based interventions, and addresses potential ethical concerns since all units receive the intervention by the end of the trial. TRUE HAVEN implements a 6-month intervention package sequentially across six neighborhoods of New Haven with high rates of gun violence and evaluates the intervention’s impact over extended follow-up periods (see Table 1 for the timeline).

**Table 1:** Timeline of TRUE HAVEN and list of activities by year and 6-month period

	Year 1		Year 2		Year 3		Year 4		Year 5	
**Activity**	1–6	7–12	13–18	19–24	25–30	31–36	37–42	43–48	49–54	55–60
Preparing, planning, piloting, and optimizing										
Research team meetings	**•**	**•**	**•**	**•**	**•**	**•**	**•**	**•**	**•**	**•**
Hire/train research staff; IRB approval	**•**									
Convene CAB, plan and pilot interventions	**•**									
Optimize interventions; weekly data review and adaptation		**•**								
Implementation and evaluation										
Recruit families affected by incarceration, with 2 new neighborhoods randomly eligible each year (stepped wedge)			**•**	**•**	**•**	**•**	**•**	**•**		
Rapid qualitative and quantitative data collection (e.g., ROMA, interviews of clients/staff)	**•**	**•**	**•**	**•**	**•**	**•**	**•**	**•**	**•**	**•**
CAB meets monthly to review data, evaluate progress, and adapt		**•**	**•**	**•**	**•**	**•**	**•**	**•**	**•**	**•**
DSMB meets bi-annually to review data and safety		**•**		**•**		**•**		**•**		**•**
Assess Final Intervention Outcomes and Disseminate										
Overall evaluation of effectiveness on study outcomes							**•**	**•**	**•**	**•**
Cost-effectiveness analyses									**•**	**•**
Use simulation to develop partnerships to plan for sustainability		**•**		**•**		**•**		**•**	**•**	**•**
Manuscript preparation and submission		**•**		**•**				**•**		**•**
Community-facing dissemination		**•**		**•**		**•**		**•**	**•**	**•**

The primary outcome is neighborhood rates of incident gun violence defined by the annualized rate of gun violence incidents out of the total adult population of the neighborhood. We will also measure secondary outcomes and other outcomes at the individual, implementer/program, and neighborhood/city levels using quantitative and/or qualitative data, where implementers are the ULSC staff responsible for the housing stability intervention component. We also employ the RE-AIM implementation science framework dimensions [25] to organize these multi-level outcomes assessing reach, effectiveness, adoption, implementation, and maintenance of our TRUE HAVEN intervention package. In addition, we will assess the effectiveness of each intervention component on relevant proximal outcomes, such as rates of home ownership and participant well-being, as well as their effects on other secondary outcomes at the individual, program/implementer, and neighborhood/city levels (see Table 2 for a full listing of these outcomes).

**Table 2:** Implementation and effectiveness outcomes for TRUE HAVEN intervention under the RE-AIM framework. Abbreviations: H = housing; F = financial security; T = breaking cycle of trauma; NHPD = New Haven police department; PM = project manager; CHFA = Connecticut Housing Finance Authority

			TRUE HAVEN Component	
Outcomes by RE-AIM Dimension	Level	Approach		Assessor
***Adoption***				
Acceptability, appropriateness, feasibility of intervention and strategies	Implementer	Quantitative	All	PM
# implementers approached, adopted, and agreed to participate in the intervention	Implementer	Qualitative	All	Investigators
Characteristics of adopting sites	City/Region	Quantitative	H	PM/Implementers
% lenders requiring comprehensive financial education	City	Quantitative/Mixed	F	PM/Implementers
# of orgs requesting trauma-informed care curriculum	Implementer	Quantitative/Mixed	All	PM/Investigators
***Implementation***				
Fidelity to intervention and strategies	Implementer	Quantitative/Mixed	All	PM/Investigators
Cost of intervention and strategies	Implementer	Quantitative	All	Implementers/ Investigators
# and % of individuals/families that complete program	Individual	Quantitative	All	PM/Implementers
New policies/practices that reduce barriers for families	Implementer	Quantitative/Mixed	All	Implementers/ Investigators
***Reach***				
Reach to eligible population	Individual/Family	Quantitative	All	PM/Implementers
Representativeness of participants	Individual/Family	Quantitative/Mixed	All	PM/Implementers
# counseled by trauma-informed community member	Individual/Family	Quantitative	T	Implementers
***Effectiveness***				
Acceptability/appropriateness of intervention/strategies	Individual/Family	Quantitative/Mixed	All	PM/Investigators
Participant self-efficacy	Individual/family	Quantitative/Mixed	All	PM/Implementers
% Housing costs < 30% income	Individual/Family	Quantitative	H	Implementers
% Improved credit score	Individual/Family	Quantitative	F	Implementers
$ Saved by participants	Individual/Family	Quantitative	F	Implementers
Perceived health and well-being	Individual/Family	Quantitative/Mixed	W	PM/Implementers
% Homeownership	Neighborhood	Quantitative	H	CHFA
% Denied home loan	Neighborhood	Quantitative	H	CHFA
Reason for home loan denial	Neighborhood	Quantitative	H	CHFA
Collective well-being	Neighborhood	Quantitative	T	DataHaven
Experience of discrimination	Neighborhood	Quantitative	T	DataHaven
Social cohesion	Neighborhood	Quantitative	T	DataHaven
Exposure to gun violence	Neighborhood	Quantitative	All	DataHaven
Incident gun violence	Neighborhood	Quantitative	All	NHPD
***Maintenance***				
Maintenance of fidelity and delivery	Implementers	Quantitative/Mixed	All	PM/Implementers
New structures, policies, practices and values that address structural racism	Neighborhood/ City	Quantitative/Mixed	All	Implementers/ Investigators
# and % participants that remain in their home for > 2y	Individual/family	Quantitative	H	Implementers
% families/individuals evicted or homes foreclosed	Neighborhood	Quantitative	H/F	CHFA
$ amount of home loans administered each year	Neighborhood	Quantitative	H	CHFA
# and % participants that kept job for > 2 years	Individual/family	Quantitative	F	Implementers
Social cohesion trend	Neighborhood	Quantitative	T	DataHaven
Yearly incidence of gun violence trend	Neighborhood	Quantitative	All	NHPD
Collective well-being trend	Neighborhood	Quantitative	All	DataHaven

Following the principles of community-based participatory research, all aspects of this project -- from study design, through resource governance, and implementation -- have been achieved through collaborating with community partner organizations. A Community Advisory Board (CAB) that is comprised of racially and ethnically diverse community members living in neighborhoods with high rates of gun violence, firearm assault survivors, formerly incarcerated people, leaders of community-based organizations, educators, academics, city officials, youth mentoring programs, violence deterrence programs, probation officers, and physical and mental healthcare and other social service providers, has been overseeing and guiding this work for more than a decade. Coordinated by the Urban League of Southern Connecticut (ULSC), a regional civil rights organization that aims to empower economically disadvantaged people and groups to secure and sustain economic self-reliance and parity among communities in Connecticut, the CAB meets monthly and will deliver or support delivery of relevant intervention components, as listed in Supplemental Table 1.

### Study context and institutional review

New Haven, CT, is a small city that, like most cities in the US, experiences significant neighborhood-level health inequities. There is an approximate 12-year gap in life expectancy between the low-income, Black/Latinx neighborhoods that had been redlined as Grade D neighborhoods decades ago, where banks systematically denied loans for homes and businesses, compared with those that were rated Grade A (see Supplemental Fig. 1) [26]. These neighborhoods also experience higher rates of gun violence, incarceration, unemployment, and housing instability [1]. Mean rates of gun violence between 2011 and 2020 in our six predominantly Black/Latinx neighborhoods range from 70 to 248 shootings per 100,000 population, compared with 15–38 shootings per 100,000 population in non-redlined neighborhoods and 4.4 shootings per 100,000 population nationally. Our team has recognized that large-scale, community-wide, systemic interventions are needed to curb the incidence and effects of gun violence.

TRUE HAVEN is a partnership between the ULSC and the Yale School of Medicine, specifically, Yale’s Center for Research Engagement and the SEICHE Center for Health and Justice; the Yale Center for Methods in Implementation and Prevention Science supports intervention optimization and statistics. The ULSC will apply the framework of collective well-being to implement a multi-level intervention that leverages local partnerships to create community conditions that enable four domains of collective well-being. Coordinated by the ULSC, the partner organizations that will deliver or provide resources to support the delivery of relevant intervention components are listed in Supplemental Table 1 along with their respective roles. In addition, DataHaven, a local non-profit that implements a citywide Community Wellbeing Survey among a population-based sample, will provide neighborhood-level data on community well-being and cohesion and other neighborhood-level outcomes each year of our study; the New Haven Police Department will additionally provide monthly neighborhood-level data on gun violence throughout the study duration. Lastly, because the sustainability of this intervention depends on engaging institutions, organizations, and government entities that have benefited historically from structural racism, they will provide financial resources for down payment and rental assistance for the housing stability component of the intervention (e.g., Yale New Haven Hospital, Yale University, Yale School of Medicine, CT Department of Housing, Livable Cities Initiative, Connecticut Housing Finance Authority, and the Community Foundation of Greater New Haven).

### Intervention overview

Our community-academic team designed TRUE HAVEN, a community-based intervention that acts on two leverage points in our community’s systems that are most feasibly intervened upon and projected to be most impactful: (1) housing stability (for recently incarcerated individuals as well as family members of incarcerated people) and (2) mental well-being. We will study the effect of our intervention using a cluster-randomized, stepped-wedge study design. Each of six neighborhood’s residents will be eligible for enrollment in the housing stability component of the initiative for a 6-month period and during this time, trusted community members in this neighborhood will be trained in trauma-informed counseling. In addition, TRUE HAVEN will employ the Learn As You Go (LAGO) approach [27] that analyzes intervention implementation and outcome data at each trial stage to tweak and tailor the intervention as the trial progresses to optimize the cost-effectiveness of the package, mitigating the risk of an intervention failure. LAGO will provide rich contextual and experiential data for our evaluation of the intervention package.

#### Housing stability

TRUE HAVEN aims to mitigate the effects of structural racism by fostering housing stability and supporting home ownership among people affected by incarceration, including those that have a family member incarcerated and individuals returning to the community from prison. We will partner with local organizations to provide increased access to low-interest home loans and down payment or rental assistance to participants needing financial support. All participants receiving these financial supports will engage in a comprehensive, milestone-based financial education program through the ULSC or Neighborhood Housing Services (NHS) to learn how credit scores impact purchase power, financial terms in banking, managing unexpected expenses, preparation for an emergency expense, and developing habits to budget monthly spending. All participating organizations have agreed there will be accessing financial supports or leases or loans without considering family member’s or personal history of incarceration. We plan to support 1,400 individuals returning to the community from incarceration and/or their family members (i.e., spouse/partner, co-parent of a child, parent, sibling, or child) from these 6 neighborhoods. In addition, as part of its usual services, ULSC or NHS will also connect participants to job training and employment opportunities, as needed.

#### Mental wellness

We will partner with Clifford Beers Community Health Partners, an integrated network of care that offers “wrap around” mental health and social services to children and families, to train community members (e.g., barbers, faith leaders, youth mentors, educators) on the impact of trauma so they can recognize those affected by the trauma of gun violence and be able to properly support them, engage them in skill building to effectively regulate emotions, and connect them to formal mental health resources, if needed [3]. The purpose of the training is to offer education on trauma prevalence and research on childhood adversity as well as its potential long-term impacts; how to recognize the impact of trauma on the body, brain and behaviors and an opportunity for dialogue about various types of trauma impacting the community (acute, chronic, historical, race-based and systemic trauma) and to share common language regarding how trauma and stress affect the community’s overall wellbeing. Trainings will also include discussion on how to actively support and refer people in need of formal mental health treatment through support from partnered community health workers. We anticipate training at least 50 trusted community members from the target neighborhood in trauma-informed care and counseling each half-year. We will work with our community partners and local neighborhood groups to recruit participants for these trainings. We aim to recruit a diverse group of community members, including youth mentors, barbers, faith leaders, teachers, and other local influencers, who will then interact with community members as they encounter them in the course of their work and lives.

#### Coalition to address structural racism

We will convene a coalition of diverse stakeholders quarterly to elicit and review barriers to achieving housing stability and accessing mental health supports for our participants and to actively and collectively work to address these barriers. This Steering Committee (SC) is inclusive of CAB Members and augments this group with additional leaders at the local, city, and state levels, such as city and state officials, local and regional philanthropy, local businesses, law enforcement, and physical and mental healthcare and other social service providers and leadership of coalitions representing these providers. The TRUE HAVEN SC is well-suited to both identify policy and programmatic barriers to achieving equitable access to stable housing and mental healthcare as well as to collaboratively find and enact solutions to address such barriers. The SC will meet quarterly, review data on ongoing study outcomes, review information on barriers that have arisen to housing stability and accessing mental healthcare, and will engage in facilitated processes to propose and enact plans to address those barriers. We will track the number of barriers identified and initiatives enacted to address them at the local, city, and state levels over time.

#### Inclusion and exclusion criteria

**Fig. 2:**
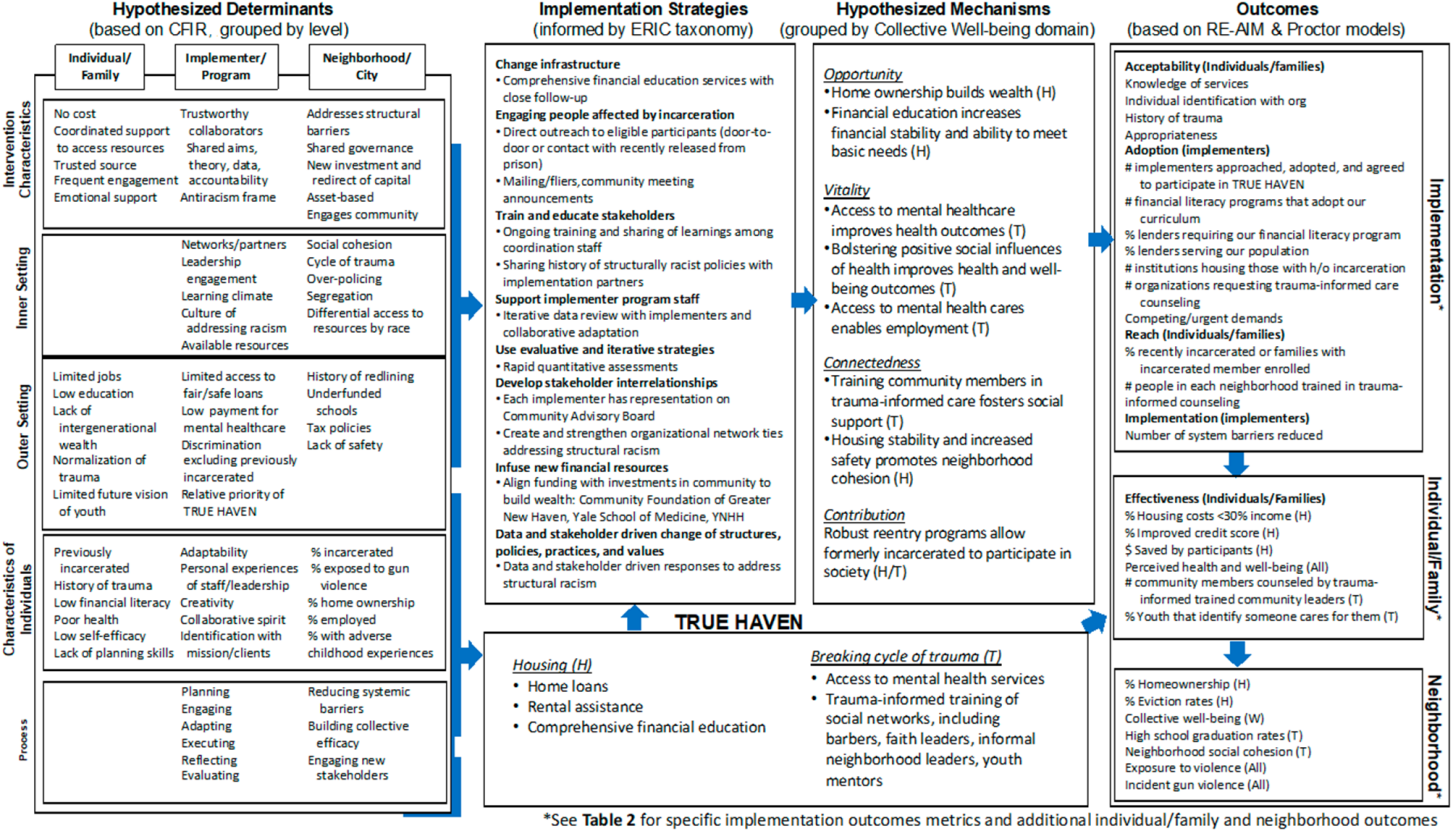
Logic model describing the multi-level, multi-component TRUE HAVEN intervention and measurable outcomes

The primary outcome of TRUE HAVEN is a community-level outcome, incidence of neighborhood gun violence, and, as such, there are no inclusion or exclusion criteria. However, for the housing assistance intervention component, individuals are eligible for study entry if they: (1) live in one of the six neighborhoods with highest rates of gun violence in New Haven, CT, including Hill North & South, Fair Haven, Newhallville, Dixwell, West River/Dwight and Beaver Hills/Edgewood (Fig. 1); (2) are over 18 years of age; (3) were previously incarcerated within the last 12 months or have a family member who is currently incarcerated (i.e., spouse/partner, co-parent of shared child, sibling, parent, child). The rationale for these criteria is to target people who are at high risk of eviction and social instability when their family unit has been disrupted by incarceration, which is associated with involvement in gun violence. Individuals are excluded if they plan to leave the study area within 1 year. This exclusion criterion excludes participants who are unable to complete follow up and thus unable influence or be influenced by neighborhood changes. Concomitant interventions are permitted.

#### Recruitment, procedures, and follow-up

For the housing stability component, the recruitment and assessment processes are described above and summarized in Fig. 2. The hypothesized determinants were originally developed based on the Consolidated Framework for Implementation Research (CFIR) and grouped by levels [28]. Participants meeting the inclusion criteria and who consent to participate will complete a baseline assessment on the day of enrollment. Participants will then engage in a comprehensive, tailored financial education and counseling program through the ULSC to address their housing instability. The education component includes learning objectives such as understanding how credit scores work, creating and maintaining a budget, developing habits to save money, setting sound financial goals, and avoiding debt. The financial education program is milestone-based, so participants move forward to the next lesson only after prior content has been mastered. This approach fosters self-efficacy and confidence and ensures graduates of the program are equipped with the skills needed to maintain financial security after financial supports expire. Financial supports in the form of rental assistance or down payment assistance and/or low-interest home loans will be provided to participants when the appropriate milestones have been achieved. Most people are able to complete the financial education program within six months. At the beginning and end of their course, participants will complete a knowledge assessment to assess their understanding of financial topics (e.g., how credit reports work and how much money should be in savings) and their perceptions of the acceptability, appropriateness, and feasibility of the program. At the time of enrollment and at 6-months, 1-year and 2-years, ULSC will contact participants by phone to assess their housing situation (i.e., they remained in their home/apartment, moved to a different home/apartment and if so why, were evicted, underwent foreclosure) and financial situation (i.e., how much in savings). We will also assess other outcomes such as self-efficacy, perceived health and well-being, and financial knowledge.

In addition, a subset of participants in each neighborhood will be asked if they are willing to participate in a focus group to explore implementation outcomes (e.g., satisfaction with the intervention and content delivery, perceived usefulness of the intervention) in the second half of or right after their 6-month enrollment period. These focus groups will include a purposive sample of 20 participants each half year, stratified by gender, race, and age, and each focus group will have no more than 8 participants. We will hold separate focus groups with men and women to understand if and how various factors affect men and women differently. The focus group will be no longer than 90 min in length and participation will be voluntary – the participants will still be able to complete the other parts of the program if they elect not to join the focus group. We will also collect data on implementation outcomes (i.e., adoption, acceptability, feasibility, appropriateness) from the staff members delivering the financial education (i.e., housing counselors; other ULSC staff). This will be in the form of bi-annual assessments programmed in Qualtrics and administered by the program manager of this study in person or over the phone. We will also recruit a sample of staff for focus groups yearly to qualitatively explore these outcomes. Participant dropout and retention will be monitored.

The above procedures all refer to the housing assistance part of the intervention. In addition, we will also recruit 50 community members from each neighborhood to be trained in trauma-informed counseling by our partners at Clifford Beers Clinic. We will ask them to track and report the number of people they counsel each week to the program manager. Some of them may be asked to participate in implementer focus groups as well. Outcomes will be assessed in aggregate at the neighborhood level, including how many people were newly referred for mental health care and neighborhood rates of depression, anxiety, and symptoms of post-traumatic stress. Still, we will not be obtaining data directly from the individuals who are counseled or seek mental health care.

We train personnel at ULSC in informed consent procedures and confidentiality. All surveys will be administered by an intake specialist at ULSC, who has completed training that includes: (a) basic interviewing techniques, (b) human subject rights, (c) issues of confidentiality and anonymity, and (d) details of the survey instrument. Interviewer training will be a detailed process to ensure that the purposes and sponsorship of the research are explained thoroughly, that questions are phrased in a non-threatening manner, that the procedures for confidentiality are clearly understood, and that interviewers are skilled in communicating these facts to potential respondents. Prior to working on the project, the interviewer will sign an agreement of confidentiality prohibiting any inappropriate use of information as outlined in this proposal, institutional review board (IRB) standards, or the Certificate of Confidentiality.

### Neighborhood-level randomization

**Fig. 3:**
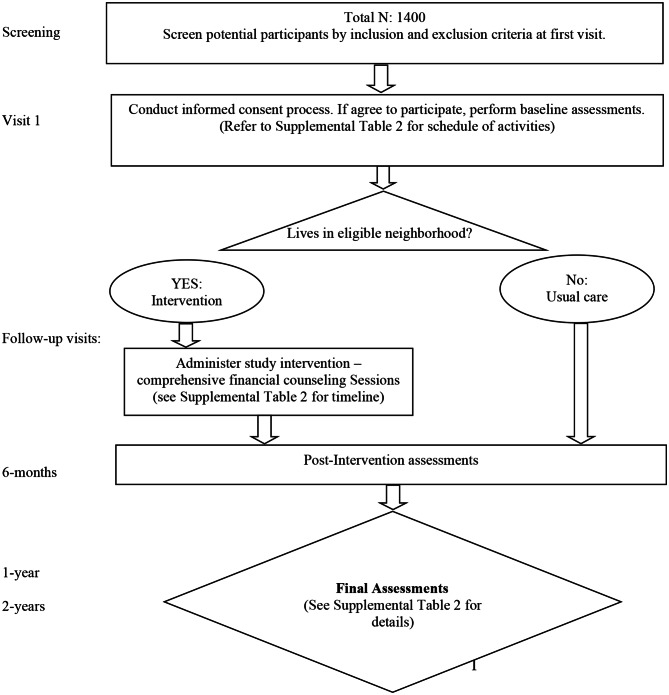
Flow diagram for the recruitment and assessment of participants in the housing assistance component of TRUE HAVEN.

**Fig. 4:**
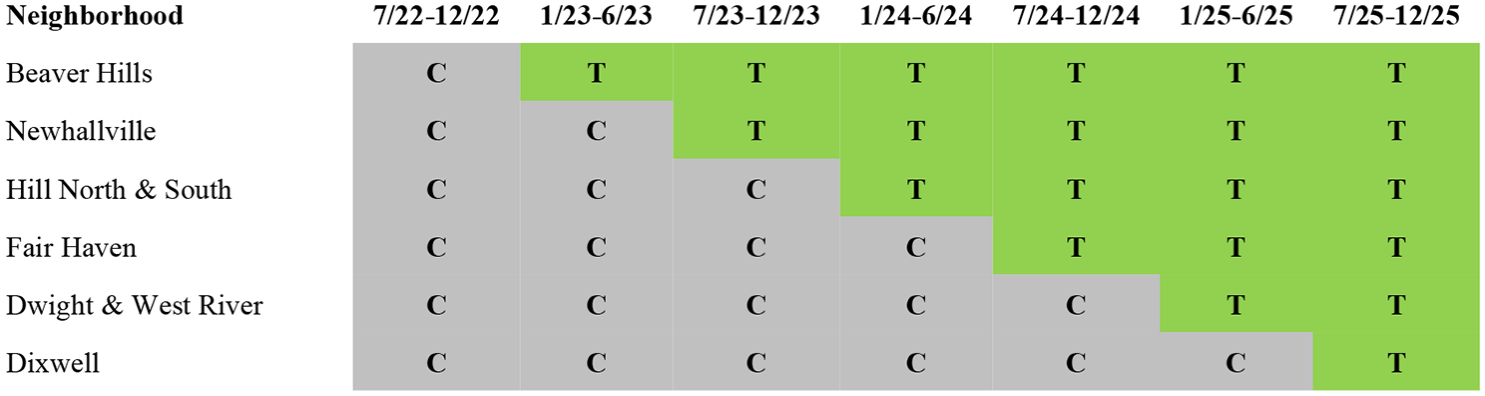
Schematic diagram for the community-level randomization (T: treatment period; C: control period)

Neighborhood-level randomization was carried out by the TRUE HAVEN CAB on August 5th, 2022 by randomly drawing names without replacement of the six participating neighborhoods from a box. While overseeing and monitoring the implementation of TRUE HAVEN, CAB members are also part of the intervention as implementers and/or as part of the larger SC responsible for changing discriminatory policies and lowering barriers to achieving housing stability and accessing mental health. They will also be the decision-makers on program adaptations. The schematic diagram for the randomization is in Fig. 3, following the Consolidated Standards of Reporting Trials (CONSORT) extension for the stepped wedge cluster randomized trials [29] (see Supplemental File 2 for completed CONSORT checklist) and Standard Protocol Items: Recommendations for Interventional Trials (SPIRIT) [30] (see Supplemental File 3 for the completed SPIRIT checklist). The SPIRIT diagram is presented in Fig. 4.

### Outcome measures and data collection

#### Primary outcome

The primary outcome of this study is the neighborhood incident gun violence rate. These administrative data will be provided monthly by the New Haven Police Department. This outcome is assessed at the neighborhood level.

#### Secondary outcomes

The first secondary outcome is neighborhood social cohesion, which is measured by the Social Cohesion sub-scale of the Sampson Collective Efficacy Scale [31]. It is a neighborhood-level outcome on residents’ perception of the strengths of relationships and solidarity among their neighbors. Social cohesion and additional neighborhood-level rates of depression, anxiety, and symptoms of post-traumatic stress will be provided by DataHaven yearly from a representative sample. The other secondary outcomes are measured among participants in the housing stability component, and include (1) participant self-efficacy: participant belief in their capacity to execute behaviors necessary to achieve goals, measured by the Generalized Self-Efficacy Scale [32]; (2) perceived health and well-being, to be assessed by the 100 Million Healthier Lives Adult Well-being Assessment [33] including measures on subjective well-being, general health, health problems, sense of direction and purpose in life, emotional support, and sense of belonging to community; (3) financial security, assessed by financial readiness for an emergency. These participant-level secondary outcomes will be measured every six months.

#### Implementation outcomes

Following the RE-AIM implementation science framework [25], assessments on implementation outcomes are collected by research coordinators at baseline and every six months following the initiation of the intervention in each neighborhood. We will use validated scales to assess implementation outcomes, including measures of acceptability, appropriateness, and feasibility by Weiner, et al [18], and measures of fidelity and reporting adaptations and modifications following the Framework for Reporting Adaptations and Modifications-Enhanced (FRAME) [34].

#### Data collection

For the primary outcome, the New Haven Police Department will provide neighborhood-level data on gun assaults. DataHaven, a not-for-profit organization that collects neighborhood-level data on community wellbeing and social cohesion, is our key community partner who will provide neighborhood-level outcomes each year of our study. Connecticut Housing Finance Authority (CHFA) will provide neighborhood-level data on home ownership, loan denial, eviction and foreclosure. In addition, for participants recruited to the housing assistance program, information on sociodemographic backgrounds will be collected at the baseline, and questionnaires will be administered verbally either by phone or in-person at the 6-month visit by a housing counselor, and at the 1-year and 2-year time points by phone by the program manager. For qualitative and implementation outcomes, there may be additional *de novo* questions added that stakeholders think are important to include. Likewise, focus group questions will be developed in collaboration with our CAB and implementation partners and submitted to the IRB for approval prior to holding any focus groups.

#### Implementation-focused process evaluation

**Fig. 5:**
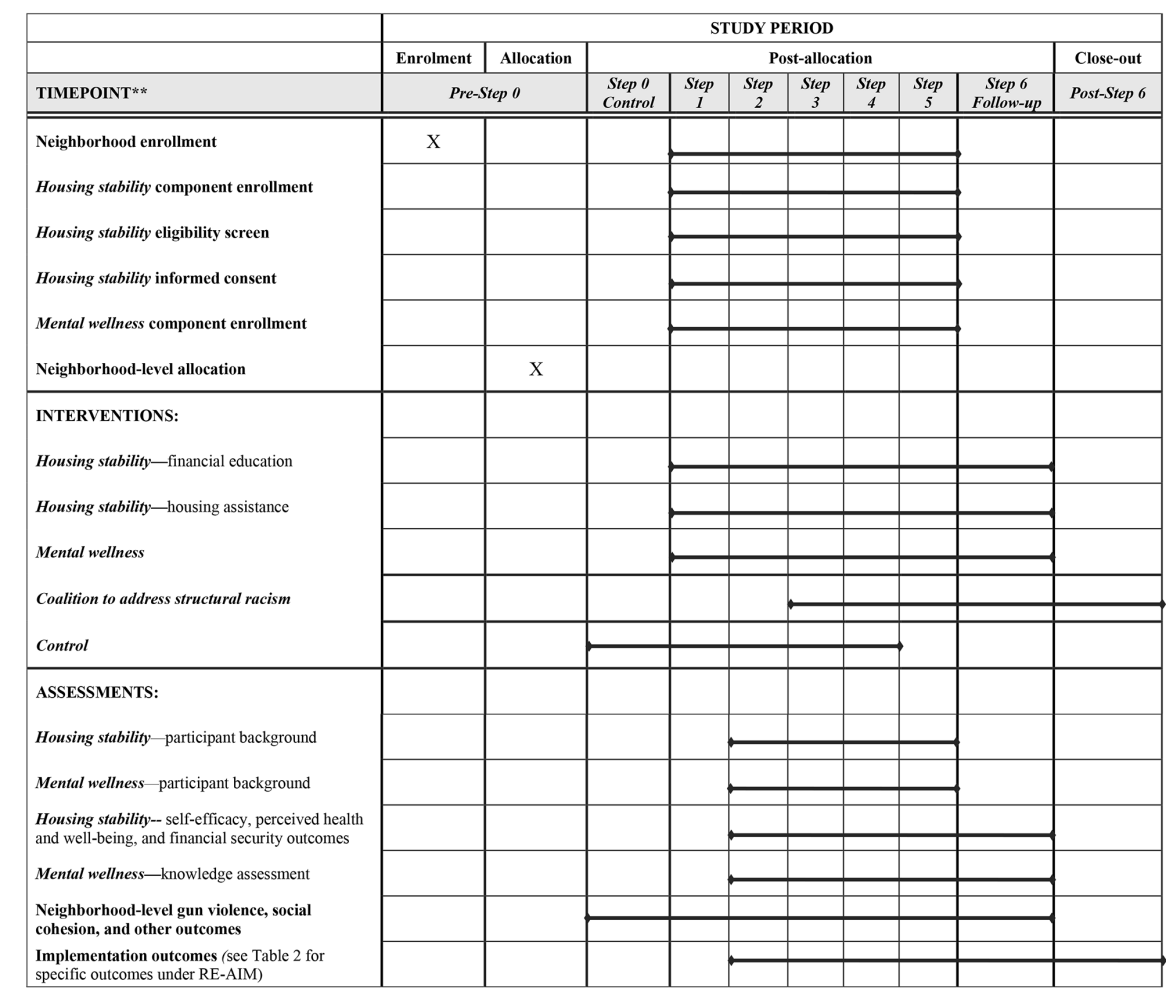
Standard Protocol Items: Recommendations for Interventional Trials (SPIRIT) flow diagram for TRUE HAVEN.

Our implementation-focused process evaluation of TRUE HAVEN, grounded in the RE-AIM [3] and Proctor [35] frameworks, will include minutes from CAB and steering committee meetings, minutes and logs from study team meetings, site visits, as well as interview data collected from enrolled participants. The logic model describing the multi-level, multi-component TRUE HAVEN intervention and measurable outcomes are presented in Fig. 5. Protocol modifications, if needed, will be guided by FRAME [34]. We will assess the following domains: (1) fidelity to intervention and strategies; (2) cost of interventions and strategies; (3) number and percentage of individuals that complete program; (4) number of services coordinated by each coordinator; (5) number of services coordinated for each family; and (6) new policies/practices that reduce barriers for families.

### Statistical considerations

#### Justification of sample size

The primary aim of this study is to compare the intervention effect of TRUE HAVEN versus control on average yearly shooting victim rates. With an average of 10,295 residents per neighborhood per year, leading to approximately 432,390 person-steps of follow-up over 3.5 years, for a two-sided test between Poisson rates with a Type I error of 0.05, intra-cluster correlation coefficient (ICC) of 0.50 as currently estimated in these 6 neighborhoods of New Haven, and a baseline shooting rate of 6 shooting victims per 10,000 persons per step in the control period as currently observed on average in these New Haven neighborhoods, our study will have about 80% power to detect a 28% decrease or greater in the primary outcome, average neighborhood shooting rates, in relation to the full TRUE HAVEN intervention package [24, 36].

For individual-level outcomes of the housing assistance component, with approximately 1400 total participants (i.e., 233 participant individuals/families per neighborhood group) enrolled during the 3.5 year study period, that is, about 33 participants per neighborhood per 0.5 year step, for binary outcomes (e.g., % homeownership, % housing costs < 30% of income, % improved credit score), for a two-sided test of the difference between proportions with a Type 1 error of 0.05, with ICCs as low as 0.01 and as high as 0.2, our study will achieve at least 80% power to detect an increase with the TRUE HAVEN intervention of at least 2.4% points for outcomes where the baseline proportion is 1% or less, 10% points for outcomes where the baseline proportion is 20% or less, and 12% points for outcomes where the baseline proportion is 40% or less.

#### Statistical analyses

For the primary outcome, we will use a log-Poisson mixed effects model for the neighborhood shooting rates $\text{log}\left[\text{E}\left({Y}_{ij}=1|{\varvec{X}}_{ij}\right)\right]= {\beta }_{0} + {\beta }_{1}{X}_{ij}+{\varvec{\beta }}_{2}^{{\prime }{\varvec{t}}_{ij}}+{N}_{i}+ {b}_{i}$, where ${Y}_{ij} \text{i}\text{s}$ the number of individuals affected by gun violence for the *ith* neighborhood, *i = 1,…6*, at the *jth* step, *j = 1,…,7*,${X}_{ij}$is the intervention status for the $i$^*th*^ neighborhood at the $j$^*th*^ step, ${\varvec{t}}_{ij}$ is an indicator function for the step number, ${N}_{i}$ is the offset corresponding to the population of the $i$^*th*^ neighborhood, and ${b}_{i}$ is a mean-zero random neighborhood effect. $\text{e}\text{x}\text{p}\left[{\beta }_{1}\right]$ is the parameter of interest, the rate ratio for the overall intervention effect and the natural exponent of the elements of ${\varvec{\beta }}_{2}$ are the rate ratios for the 6 step time effects. A generalized estimating equations approach [37] will be taken for estimation and inference will use the robust score test, and an independence working correlation matrix to account for correlations within neighborhoods in inference. Many secondary analyses of the primary endpoint can be conducted. With 6 neighborhoods, residual confounding is possible, and in secondary analysis, the model will include adjustment for potential neighborhood-level confounders, such as median income, percent unemployed, and population density. Secondary analysis of the primary outcome will consider modification of the intervention effect by pre-specified potential modifiers, such as race/ethnicity composition of the neighborhood, median neighborhood income and season. These interactions will be assessed by robust score tests of the suitable cross-product terms.

Further secondary analysis will involve the specific outcomes of the two key components of TRUE HAVEN -- housing and mental health services for families affected by incarceration. Each of these components acts at either the neighborhood, program/implementer or individual/family levels and will include different populations with different sample sizes. In general, the overall approaches to analysis described above will be taken. When outcomes are continuous, generalized estimating equations will typically use the identify link function, and when they are binary, they will use the log link function. If the outcomes are repeated within unit over time, a second random effect will be included to induce the correlation due to repeated measures within, say, individuals or implementers. Mediation analysis will be another informative feature of the statistical analysis plan. Here, we will assess the extent to which any improvements to neighborhood shooting rates are mediated by beneficial changes in downstream process outcomes due to the intervention. These process outcomes could include those given by the RE-AIM and Proctor frameworks in the project logic model presented in Fig. 2. Mediation analysis [38, 39] for downstream effects will use the difference method. Inverse probability weighting will be used to address missingness in outcomes, while missing indicator methods will be used to handle missing covariates when applicable.

### Protection of participants

The risks associated with participating in this study can be categorized as minimal (i.e., risks are regarded as being similar to everyday risks, and there is adequate surveillance and protections integrated into the ongoing evaluation plan to discover adverse events promptly and keep their effects minimal). We will use procedures to promptly detect and respond to adverse events so as to minimize their effects. Nonetheless, we will convene a Data and Safety Monitoring Board (DSMB), a three-person team with expertise in statistical analysis and public health research among marginalized populations. DSMB will convene to (1) periodically review and evaluate the accumulated study data for participant safety, study conduct and progress, and, when appropriate, effectiveness, and (2) make recommendations to National Institute of Health concerning the continuation, modification, or termination of the trial. The DSMB considers study-specific data as well as relevant background knowledge about the intervention and population under study. Prior to initiating any data review, the DSMB will be responsible for defining its deliberative processes, including: event triggers that would call for an unscheduled review, stopping procedures that are consistent with the protocol, and voting procedures. The DSMB is also responsible for maintaining the confidentiality of its internal discussions and activities as well as the contents of reports provided to it.

The DSMB will review the protocol for any major concern prior to implementation. During the study period, the DSMB will review cumulative study data to evaluate safety, study conduct, and scientific validity and integrity of the trial at 6-month intervals. As part of this responsibility, DSMB members must be satisfied that the timeliness, completeness, and accuracy of the data submitted to them for review are sufficient for evaluation of the safety and welfare of all study participants. The DSMB will also assess the performance of overall study operations and any other relevant issues, as necessary.

### Data input, storage and management

Survey responses collected electronically through Yale Qualtrics and data transferred from collaborative organizations (e.g., New Haven Police) will be stored on Yale University encrypted, password-protected computers and servers. All community-based organizations delivering the interventions have the necessary authorizations to collect and store the relevant data (e.g., Housing and Urban Development certification). All paper forms with identifiable information will be securely stored in a locked cabinet in the Yale SEICHE Center for Health and Justice until the completion of the study. Once the data from various administrative databases have been linked, the dataset will be stripped of individual identifiers prior to data analyses. Data analyses will be carried out by study analysts, and data access will be limited to only authorized study team members. At the end of the study, all records will continue to be kept in a secure location for as long a period as dictated by the reviewing IRB, institutional policies, regulatory, or sponsor/funding agency requirements.

### Current status of TRUE HAVEN

TRUE HAVEN opened for enrollment on February 1, 2023, and is ongoing for participant recruitment (see the sample flyer in Supplemental File 5). Our study team holds regular meetings, site visits to local partner organizations to promote recruitment, and trainings. The CAB holds monthly meetings and the SC holds quarterly meetings and is comprised of representatives from each of the implementation partner organizations (see Supplemental Table 1) and also members of complementary organizations working to reduce rates of gun violence in New Haven, such as Project Longevity, a program that uses community involvement, social services, and focused policing to positively reduce involvement in gun violence. All community partners are paid for their time. For in person interactions with participants or research staff, we ensure study procedures focus on minimizing risk of COVID-19 transmission with mask-wearing and optimized social distancing (6 feet), and maintaining flexibility in schedule for any uncertainty with the circumstances, such as COVID infection among participants or implementing staff.

## Discussion

TRUE HAVEN is a novel multi-level intervention package that aims to reduce rates of community gun violence through three strategies that focus on increasing housing stability (individual/family level), mental well-being (neighborhood/community level), and addressing structurally racist policies (city/state level) in New Haven, CT. Several aspects make our study innovative. First, our study applies a novel theoretical framework of collective well-being to upend the effects of centuries of structural racism that has resulted high prevalence of gun violence. This differs from traditional approaches to gun violence that focus on the victim in isolation or on remediating the perpetrator of gun violence, as we recognize that the family and community context has a greater long-term influence on behaviors and outcomes.

Second, we design this multi-system strategy to strengthen the community’s self-sufficiency across multiple domains of collective well-being. We recognize that single-system approaches like supporting housing or increasing access to mental healthcare alone are insufficient to sustain positive outcomes because of other counteracting community-level forces.

Third, we apply advanced and novel methods in implementation science and experimental design to study the effectiveness and impact of the intervention package. We embed this research in an implementation science framework to systematically assess whether change in processes (e.g., convening a community advisory board) and progress towards achieving outcomes related to addressing structural racism (e.g., percent homeownership and number of racist policies changed), reduces the rates and effects of gun violence, and improves the health and overall well-being of all in our community. We employ a stepped wedge design that sequentially randomizes the six neighborhoods with among the highest rates of gun violence in the New Haven Area. Our community-based implementation science approach, coupled with the stepped wedge design, will be the first time such a design is used to evaluate an intervention package on gun violence through addressing structural racism in an integrated manner. We will collect multi-level data at the implementer level, individual level, neighborhood-level, and city level on the adoption, implementation, reach, effectiveness and maintenance of components of the intervention package. Such an effort can help inform future implementation efforts both in New Haven and in other community contexts.

Fourth, we have engaged institutions that have benefited from structural racism to actively re-invest in these communities. We will establish a process for strengthening and shifting the power balance towards these historically marginalized communities to realize their untapped and oppressed potential. New Haven has a community of powerful local institutions including hospitals, universities, and city leaders who have recognized *structural racism* as a public health threat and are committed to recognizing their own culpability and to repair harm through community reinvestment. Study results will be peer-reviewed, published, and communicated to participating neighborhoods and community organizations.

### Limitations

First, the neighborhoods under intervention are geographically close to each other and social networks spanning across neighborhood are expected. Benefits received from the intervention among those in one neighborhood may affect those in another, which leads to the spillover of the treatment effect. Second, TRUE HAVEN is a non-blinded study due to practical considerations, and the sequenced time of initiation of the intervention across the neighborhoods are known. Although the primary outcome of gun violence incident rate is perhaps less at risk of bias from lack of blinding, certain secondary outcomes could result in bias due to non-blinding. Third, our study site is located in New Haven, CT, a northeast U.S. city, and has potentially limited generalizability to other locations where the causes of gun violence and the amount of community resources may be different. Fourth, there may be exogenous factors that affect rates of gun violence over the course of 5 years (e.g., changes in funding, elections of new political leaders) that make the results more difficult to interpret. We can use data from neighborhoods in similar Connecticut cities, Bridgeport and Hartford, that can serve as additional comparisons to identify secular trends.

## Conclusion

TRUE HAVEN will generate data on the effect of a novel intervention package on reducing community gun violence. At this project’s completion, evidence for whether and how addressing structural racism citywide by increasing housing stability and home ownership, income and wealth, and access to mental health services will lead to a reduction in gun violence will be available.

### Electronic supplementary material

Below is the link to the electronic supplementary material.

Supplementary Material 1

Supplementary Material 2

Supplementary Material 3

Supplementary Material 4

Supplementary Material 5

## Data Availability

Not applicable.

## List of Abbreviations

CAB: Community advisory board
CFIR: Consolidated framework for implementation research
CHFA: Connecticut Housing Finance Authority
CONSORT: Consolidated Standards of Reporting Trials
DSMB: Data and safety monitoring board
LAGO: Learn As You Go
NHPD: New Haven police department
PM: Project manager
RE-AIM: Reach, effectiveness, adoption, implementation, and maintenance
SC: Steering committee
SPIRIT: Standard protocol items:recommendations for interventional trials
TRUE HAVEN: TRUsted rEsidents and Housing Assistance to decrease Violence Exposure in New Haven
ULSC: Urban League of Southern Connecticut
