# Supplementary material for: Trusted residents and housing assistance to decrease violence exposure in New Haven (TRUE HAVEN): a strengths-based and community-driven stepped-wedge intervention to reduce gun violence
Source: BMC Public Health. 2023 Aug 14;23:1545. doi: 10.1186/s12889-023-15997-x (PMC10426138; doi:10.1186/s12889-023-15997-x)
Supplement: Supplementary file 1 — Supplementary Material 1 [file 12889_2023_15997_MOESM1_ESM.docx]

**Supplemental File 1. Additional tables and figures.**

|  | | |  |
| --- | --- | --- | --- |
| **TRUE HAVEN Intervention Component** | **Partner Organization** | **Role** | |
| Housing assistance | Connecticut Housing Finance Authority | Low-interest home loans and down payment assistance | |
|  | Connecticut Department of Housing | Rental assistance | |
|  | Livable City Initiative | Down payment assistance, home improvement grants | |
|  | Yale New Haven Health System | Low-interest home loans and down payment assistance | |
|  | Yale University | Low-interest home loans and down payment assistance | |
|  | Urban League of Southern Connecticut | Comprehensive financial education | |
|  | Neighborhood Housing Services | Comprehensive financial education | |
|  | Elm City Communities (Housing Authority) | Identify families ready to move out of public housing | |
| Training in trauma-informed counseling | Clifford Beers Clinic | Will train community members in trauma-informed counseling | |
|  | YCCI-Cultural Ambassadors | Network of faith leaders and leaders of other community organizations | |
|  | Barbers | Network of barbers with clients in our target neighborhoods | |

**Supplemental Table 1.** TRUE HAVEN community-based implementation partners and their roles.

|  | Recruitment | Screening | Baseline assessment | Visit 2  Day 14 ±14 | Visit 3  Day 28 ±14 | Visit 4  Day 42 ±14 | Visit 5  Day 56 ±14 | Visit 6  Day 84 ±14 | Visit 7  Day 98 ±14 | Visit 8  Day 112 ±14 | Visit 9  Day 126 ±14 | Visit 10  Day 138 ±14 | 6-month  Visit and assessment | 1-year follow-up phone call or visit | 2-year follow-up phone call or visit |  |
| --- | --- | --- | --- | --- | --- | --- | --- | --- | --- | --- | --- | --- | --- | --- | --- | --- |
| New neighborhood becomes eligible for TRUE HAVEN intervention | X |  |  |  |  |  |  |  |  |  |  |  |  |  |  |  |
| Informed Consent |  | X | X |  |  |  |  |  |  |  |  |  |  |  |  |  |
| Demographics |  |  | X |  |  |  |  |  |  |  |  |  |  |  |  |  |
| Financial knowledge |  |  | X |  |  |  |  | X |  |  |  |  | X |  |  |  |
| Intervention Delivery |  |  |  |  |  |  |  |  |  |  |  |  |  |  |  |  |
| Financial counseling sessions |  |  |  | X | X | X | X | X | X | X | X | X |  |  |  |  |
| Outcome Evaluation |  |  |  |  |  |  |  |  |  |  |  |  |  |  |  |  |
| Acceptability, appropriateness, and feasibility of intervention |  |  |  |  |  |  |  | X |  |  |  |  | X |  |  |  |
| Financial knowledge |  |  | X |  |  |  |  | X |  |  |  |  | X | X | X | |
| Self-efficacy |  |  | X |  |  |  |  |  |  |  |  |  | X | X | X | |
| Perceived health and well-being |  |  | X |  |  |  |  |  |  |  |  |  | X | X | X | |
| Housing status |  |  | X |  |  |  |  |  |  |  |  |  | X | X | X | |
| Financial status |  |  | X |  |  |  |  |  |  |  |  |  | X | X | X | |
| Adverse Events Reporting |  |  | X | X | X | X | X | X | X | X | X | X | X | X | X | |

**Supplemental Table 2.** Visit schedule table for housing stability arm of intervention.

**
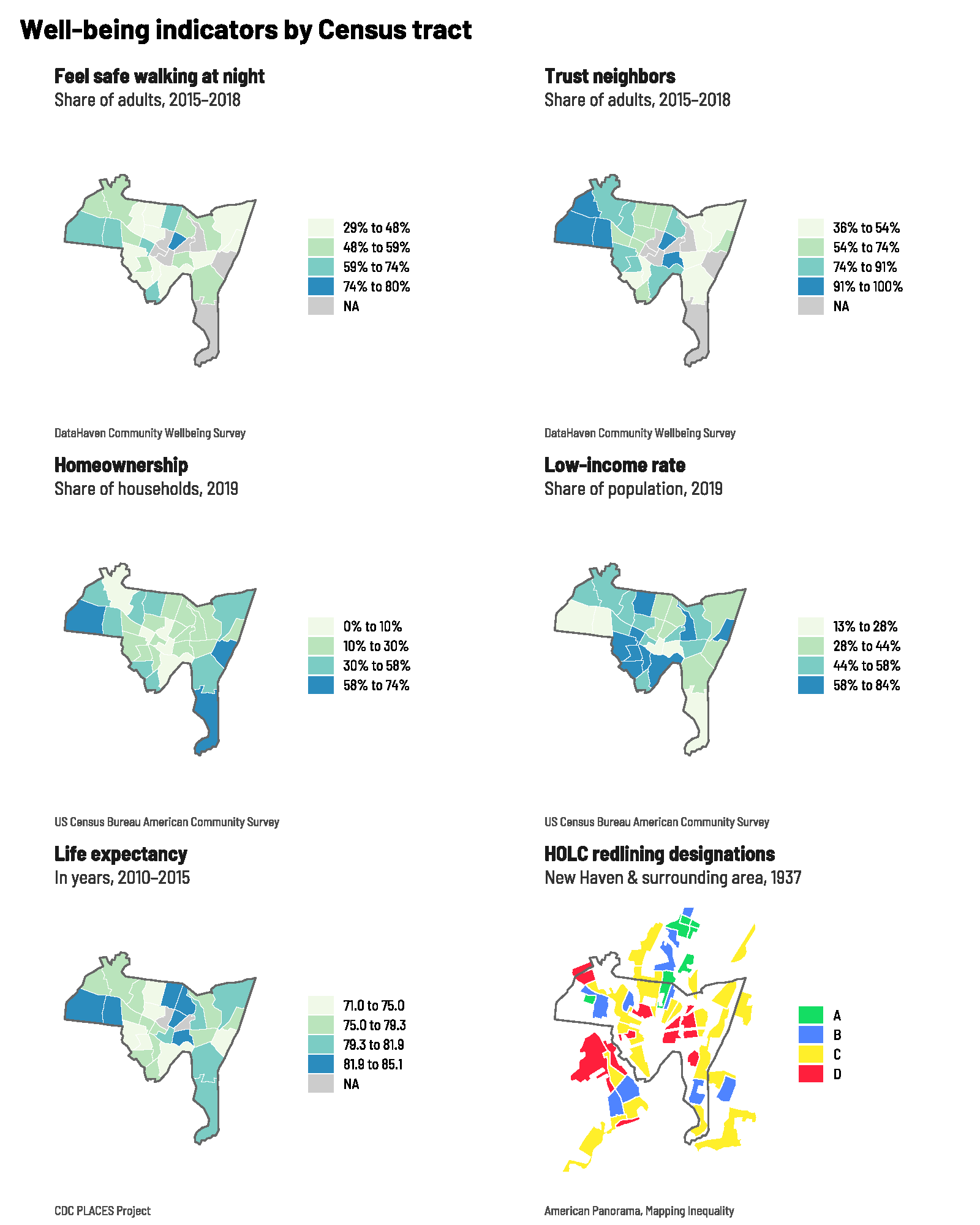
**

**Supplemental Figure 1.** Present-day indicators of health, well-being, and opportunity, as well as Home Owners Loan Corporation maps of Grade A-D neighborhoods in 1937
