# Supplementary material for: Trusted residents and housing assistance to decrease violence exposure in New Haven (TRUE HAVEN): a strengths-based and community-driven stepped-wedge intervention to reduce gun violence
Source: BMC Public Health. 2023 Aug 14;23:1545. doi: 10.1186/s12889-023-15997-x (PMC10426138; doi:10.1186/s12889-023-15997-x)
Supplement: Supplementary file 4 — Supplementary Material 4 [file 12889_2023_15997_MOESM4_ESM.docx]

### COMPOUND AUTHORIZATION AND CONSENT FOR PARTICIPATION

### IN A RESEARCH STUDY

**YALE SCHOOL OF MEDICINE & URBAN LEAGUE OF SOUTHERN CONNECTICUT**

**Study Title:** TRUE HAVEN: TRUsted rEsidents and Housing Assistance to decrease Violence Exposure in New Haven

**Principal Investigators (the people who are responsible for this research):** Dr. Brita Roy, 300 George Street, Rm. G-05, New Haven, CT 06511 and Ms. Virginia T. Spell, 136 Sherman Ave., New Haven, CT 06511

**Research Study Summary:**

- We are asking you to join a research study.
- The purpose of this research study is to explore whether improving housing security and access to trauma-informed counseling reduces the rates of gun violence in New Haven.
- Study activities will include: Attending a series of financial education sessions at the Urban League of Southern Connecticut. You will also be asked to complete some questionnaires about your financial knowledge, financial situation, empowerment, and overall health and well-being a few times over the course of 2 years.
- Your involvement will require 15 hours.
- There may be some risks from participating in this study. You may become tired from the amount of time needed to fill out the questionnaires and carry out the other evaluations. The questions will focus on financial knowledge, financial situation, and physical, mental, and social health, and could cause you to become emotionally upset. If this occurs, support and counseling will be available from the study principal investigators and other study team members as needed.
- The study may have no benefits to you. While there is no guaranteed benefit from participating in this study, participants and their families may directly benefit from the financial support given in the housing stability arm and/or from social services and counseling provided in the other arms of the study. The knowledge and information gained from this study will be instructive and beneficial to policy makers and program developers in efforts to address structural barriers to opportunity in historically disadvantaged neighborhoods. The iterative evaluation of the study increases its potential to result in long-term, sustainable change that will improve the neighborhood conditions for participants in the study and other residents of these neighborhoods.
- There are other choices available to you outside of this research. You may elect to receive the standard financial support and offerings that the Urban League of Southern Connecticut provides.
- Taking part in this study is your choice. You can choose to take part, or you can choose not to take part in this study. You also can change your mind at any time. Whatever choice you make will not have any effect on your relationship with the Urban League of Southern Connecticut or with the Yale School of Medicine.
- If you are interested in learning more about the study, please continue reading, or have someone read to you, the rest of this document. Ask the study staff questions about anything you do not understand. Once you understand the study, we will ask you if you wish to participate; if so, you will have to sign this form.

**Why is this study being offered to me?**

We are asking you to take part in a research study because you live in a neighborhood of New Haven that is currently eligible for this study and you or one of your family members has been affected by incarceration. We are looking for 1400 participants to be part of this research study.

**Who is paying for the study?**

The National Institute of Minority Health and Health Disparities is paying for this study.

**What is the study about?**

The purpose of this study is to explore whether improving housing security and access to trauma-informed counseling reduces the rates of gun violence in New Haven.

**What are you asking me to do and how long will it take?**

- If you agree to take part, your participation in this study will involve attending a series of 10 financial education sessions at the Urban League of Southern Connecticut over the next six months.
- We will ask you to complete questionnaires at 6-, 12, and 24-months after you enroll. The questionnaires will ask you about what you thought about the program, your financial situation, your housing situation, your financial knowledge and empowerment, and your overall health and well-being.
- We will also randomly select a subgroup of participants to participate in a focus group to further explore thoughts about the program. If you are selected for the focus group and decide to participate, it would take 1 hour.
- We think that the study will take a total of 14-15 hours of your time.

**Are there any risks from participating in this research?**

If you decide to take part in this study, you may become tired from the amount of time needed to fill out the questionnaires. Some of the survey questions will focus on financial situation and might cause you to become emotionally upset. If this occurs, support and counseling will be available from the study principal investigator and other study team members as needed.

There is also the possible risk of loss of confidentiality. However, we understand that information about you obtained in connection with your finances is personal, and we are committed to protecting the privacy of that information. We will protect your privacy by using a random study number to identify each participant. That is, we will assign a study number to you, and create a special “key” that has your name and study number. Dr. Roy and Ms. Spell will keep the key that identifies you to your coded information, and this key will be kept secure and available only to them or selected members of the research team. When the results of the research are published or discussed in conferences, no information will be included that would reveal your identity.

**How can the study possibly benefit me or others?**

You may benefit from taking part in this study. Participants and their families may directly benefit from the financial support given in the housing stability arm and/or from social services and counseling provided in the other arms of the study.

We hope that our results will add to knowledge that will be helpful to policy makers and program developers for efforts to lower barriers to opportunity in historically disadvantaged neighborhoods. This study has potential to result in long-term change that will improve the neighborhood conditions for participants in the study and other residents of these neighborhoods.

**Are there any costs to participation?**

You will not have to pay for taking part in this study. The only costs may include transportation and your time coming to the study visits.

**Will I be paid for participation?**

You will not be paid directly for taking part in the main part of this study, though you may receive financial assistance to support home ownership or rental assistance.

If you participate in a focus group, you will receive a $30 Bank of America pre-paid debit card. We will have to share your name, address, and telephone number with Bank of America for the ePayment. You will receive a card in the mail with the first payment. You will need to activate the card over the phone.

You are responsible for paying state, federal, or other taxes for the payments you receive for being in this study. Taxes are not withheld from your payments.

**How will you keep my data safe and private?**

All of your responses will be held in confidence. Only the researchers involved in this study and those responsible for research oversight (such as representatives of the Yale University Human Research Protection Program, the Yale University Institutional Review Boards, and others) will have access to any information that could identify you that you provide. We will share it with others if you agree to it or when we have to do it because U.S. or State law requires it. For example, we will tell somebody if we learn that you are hurting a child or an older person.

We will protect your privacy by using a random study number to identify each participant. That is, we will assign a study number to you, and create a special “key” that has your name and study number. Dr. Roy and Ms. Spell will keep the key that identifies you to your coded information, and this key will be kept secure and available only to them or selected members of the research team. When we publish the results of the research or talk about it in conferences, we will not use your name. If we want to use your name, we would ask you for your permission.

We may share information about you with other researchers for future research but we will not use your name or other identifiers. We will not ask you for any additional permission.

If you participate in a focus group, we will keep your information confidential. We ask that all focus group members not repeat any information shared during the focus group to others. However, we have no control over what happens outside of the group. Therefore, please, be aware of what you share in the group and do not share anything you hear from others outside of the group.

**What Information Will You Collect About Me in this Study?**

The information we are asking to use and share is called “Protected Health Information.” It is protected by a federal law called the Privacy Rule of the Health Insurance Portability and Accountability Act (HIPAA). In general, we cannot use or share your health information for research without your permission. If you want, we can give you more information about the Privacy Rule. Also, if you have any questions about the Privacy Rule and your rights, you can speak to Yale Privacy Officer at 203-432-5919.

The specific information about you and your health that we will collect, use, and share includes:

- Research study records
- Records about phone calls made as part of this research
- Records about your study visits
- Demographic information such as your age, race, ethnicity, gender, and neighborhood of resicence
- Information obtained during this research regarding your housing situtation (for example whether you rent or own your home or apartment, or experienced any evictions or foreclosures) and your financial situation (for example, how much you have in savings)

**How will you use and share my information?**

We will use your information to conduct the study described in this consent form.

We may share your information with:

- The U.S. Department of Health and Human Services (DHHS) agencies
- Representatives from Yale University, the Yale Human Research Protection Program and the Institutional Review Board (the committee that reviews, approves, and monitors research on human participants), who are responsible for ensuring research compliance. These individuals are required to keep all information confidential.
- Principal Investigator of the study
- Co-Investigators and other investigators
- Study Coordinator and Members of the Research Team
- Data and Safety Monitoring Boards and others authorized to monitor the conduct of the Study

We will do our best to make sure your information stays private. But, if we share information with people who do not have to follow the Privacy Rule, your information will no longer be protected by the Privacy Rule. Let us know if you have questions about this. However, to better protect your health information, agreements are in place with these individuals and/or companies that require that they keep your information confidential.

This research is covered by a Certificate of Confidentiality from the National Institutes of Health. The researchers with this Certificate may not disclose or use information, documents, or biospecimens that may identify you in any federal, state, or local civil, criminal, administrative, legislative, or other action, suit, or proceeding, or be used as evidence, for example, if there is a court subpoena, unless you have consented for this use. Information, documents, or biospecimens protected by this Certificate cannot be disclosed to anyone else who is not connected with the research except, if there is a federal, state, or local law that requires disclosure (such as to report child abuse or communicable diseases but not for federal, state, or local civil, criminal, administrative, legislative, or other proceedings, see below); if you have consented to the disclosure, including for your medical treatment; or if it is used for other scientific research, as allowed by federal regulations protecting research subjects.

The Certificate cannot be used to refuse a request for information from personnel of the United States federal or state government agency sponsoring the project that is needed for auditing or

program evaluation by the National Institute of Minority Health and Health Disparities, which is funding this project or for information that must be disclosed in order to meet the requirements of the federal Food and Drug Administration (FDA). You should understand that a Certificate of Confidentiality does not prevent you from voluntarily releasing information about yourself or your involvement in this research. If you want your research information released to an insurer, medical care provider, or any other person not connected with the research, you must provide consent to allow the researchers to release it.

**Why must I sign this document?**

By signing this form, you will allow researchers to use and disclose your information described above for this research study. This is to ensure that the information related to this research is available to all parties who may need it for research purposes. You always have the right to review and copy your health information in your medical record.

**What if I change my mind?**

The authorization to use and disclose your health information collected during your participation in this study will never expire. However, you may withdraw or take away your permission at any time. You may withdraw your permission by telling the study staff or by writing to **Dr. Brita Roy, Yale School of Medicine, 300 George Street, Rm. G-05, New Haven, CT 06511 or brita.roy@yale.edu**.

If you withdraw your permission, you will not be able to stay in this study but the care and services you get outside this study will not change. No new information identifying you will be gathered after the date you withdraw. Information that has already been collected may still be used and given to others until the end of the research study to insure the integrity of the study and/or study oversight.

**What if I want to refuse or end participation before the study is over?**

Taking part in this study is your choice. You can choose to take part, or you can choose not to take part in this study. You also can change your mind at any time. Whatever choice you make will not have any effect on your relationship with the Urban League of Southern Connecticut or Yale School of Medicine.

**Who should I contact if I have questions?**

Please feel free to ask about anything you don't understand.

If you have questions later or if you have a research-related problem, you can call or email either of the Principal Investigators, Dr. Brita Roy at (347) 377-3157 or brita.roy@yale.edu, or Ms. Virginia T. Spell at (203) 327-5810 or vtspell@ulsc.org**.**

If you have questions about your rights as a research participant, or you have complaints about this research, you call the Yale Institutional Review Boards at (203) 785-4688 or email [hrpp@yale.edu](mailto:hrpp@yale.edu).

A description of this clinical trial will be available on http://www.ClinicalTrials.gov, as required by U.S. Law. This Web site will not include information that can identify you. At most, the Web site will include a summary of the results. You can search this Web site at any time.

**Authorization and Documentation of Consent**

Your signature below indicates that you read and understand this consent form and the information presented and that you agree to be in this study.

We will give you a copy of this form.

| Participant Printed Name |  | Participant Signature |  | Date |
| --- | --- | --- | --- | --- |
| Person Obtaining Consent Printed Name |  | Person Obtaining Consent Signature |  | Date |
