# Supplementary material for: Trusted residents and housing assistance to decrease violence exposure in New Haven (TRUE HAVEN): a strengths-based and community-driven stepped-wedge intervention to reduce gun violence
Source: BMC Public Health. 2023 Aug 14;23:1545. doi: 10.1186/s12889-023-15997-x (PMC10426138; doi:10.1186/s12889-023-15997-x)
Supplement: Supplementary file 5 — Supplementary Material 5 [file 12889_2023_15997_MOESM5_ESM.pdf]

TRUsted rEsidents and Housing Assistance to decrease Violence Exposure in New Haven

TRUEHAVEN

Yale HIC protocol #2000032184

SEICHE at Yale

What is the Program?

Secure Housing

Housing assistance programs

Tailored financial education

New Haven families affected by incarceration

Families of those recently incarcerated

People returning home from prison

How Can I Participate?

Yale University is partnering with the Urban League of Southern Connecticut to conduct a research study to help those formerly involved in the justice system and their families secure and maintain stable housing.

We are looking for 1400 families to join the program.

Website: https://www.ulsc.org/truehaven-reentryservices
Email: truehaven@ulsc.org
Phone: (203) 577-8498

Or scan this QR code to access the website!
